# Financing care for Severe Stigmatizing Skin Diseases (SSSDs) in Liberia: challenges and opportunities

**DOI:** 10.1186/s12939-022-01781-7

**Published:** 2022-11-14

**Authors:** John Solunta Smith, Karin Diaconu, Sophie Witter, Stefanie Weiland, F. Zeela Zaizay, Sally Theobald, Rosalind McCollum, Karsor Kollie, Jerry Kollie, Hannah Berrian, India Hotopf, Lucas Sempe, Wede Tate, Laura Dean

**Affiliations:** 1 University of Liberia Pacific Institute of Research and Evaluations, Monrovia, Liberia; 2 Queen Margaret University, Edinburgh, UK; 3 American Leprosy Mission, Greenville, USA; 4 Action Transforming Lives, Monrovia, Liberia; 5 Liverpool School of Tropical Medicine, Liverpool, UK; 6 Ministry of Health (MoH), Monrovia, Liberia

**Keywords:** Equity, Health financing, Severe Stigmatizing Skin Diseases, Neglected tropical diseases, Integrated case management, Liberia

## Abstract

**Introduction:**

Neglected tropical diseases (NTDs) are an important global health challenge, however, little is known about how to effectively finance NTD related services. Integrated management in particular, is put forward as an efficient and effective treatment modality. This is a background study to a broader health economic evaluation, seeking to document the costs of integrated case management of NTDs versus standard care in Liberia. In the current study, we document barriers and facilitators to NTD care from a health financing perspective.

**Methods:**

We carried out key informant interviews with 86 health professionals and 16 national health system policymakers. 46 participants were active in counties implementing integrated case management and 40 participants were active in counties implementing standard care. We also interviewed 16 patients and community members. All interviews were transcribed and analysed using the thematic framework approach.

**Findings:**

We found that decentralization for NTD financing is not yet achieved – financing and reporting for NTDs is still centralized and largely donor-driven as a vertical programme; government involvement in NTD financing is still minimal, focused mainly on staffing, but non-governmental organisations (NGOs) or international agencies are supporting supply and procurement of medications. Donor support and involvement in NTDs are largely coordinated around the integrated case management. Quantification for goods and budget estimations are specific challenges, given the high donor dependence, particularly for NTD related costs and the government’s limited financial role at present. These challenges contribute to stockouts of medications and supplies at clinic level, while delays in payments of salaries from the government compromise staff attendance and retention. For patients, the main challenges are high transportation costs, with inflated charges due to fear and stigma amongst motorbike taxi riders, and out-of-pocket payments for medication during stockouts and food/toiletries (for in-patients).

**Conclusion:**

Our findings contribute to the limited work on financing of SSSD services in West African settings and provide insight on challenges and opportunities for financing and large costs in accessing care by households, which is also being exacerbated by stigma.

## Introduction

Severe Stigmatizing Skin Diseases (SSSDs) are recognized as skin diseases and a sub-group of NTDs which mainly affect poor and rural dwellers [1]. Globally, 1.1 billion people encountered SSSDs from more than 55 countries, including Liberia [2]. NTDs received relatively little attention until the 2012 World Health Organization (WHO) RoadMap for the Implementation for Global NTDs control, and the London Declaration on Neglected Tropical Diseases [3]. Since then, advancements have been made, with countries investing in the distribution of mass drug administration (MDA) to at-risk people and attempts to integrate case-management of NTDs into primary care, as recommended by the WHO [4, 5].

SSSDs present with skin manifestations and are associated with life-long disability, mental health issues and stigmatization [6]. For the purposes of this study, five SSSDs will be studied: leprosy, buruli ulcer (BU), yaws, lymphatic filariasis (LF) and onchocerciasis. Reducing the burden of Severe Stigmatising Skin diseases (REDRESS) is a multidisciplinary research platform used to harmonize efforts and bring partners together to engage SSSDs research [7]. SSSDs require similar approaches to case detection and management as other NTDs, presenting opportunities for integration into standard health services and improving cost–effectiveness, community awareness and surveillance, through training healthcare workers and community leaders [8]. The WHO’s NTD road map for 2030 declared that the goal of the integrated approach is to reduce morbidity, disability, and other impacts of skin NTDs [8].

### The SSSD situation in Liberia

Liberia is one of many African countries where SSSDs are endemic; across all counties, the burden of SSSDs, namely yaws, onchocerciasis, leprosy and LF (and resulting hydrocele and lymphoedema) is high among the general population [1, 6]. In Liberia, a 2012 nationwide SSSD assessment found that 258 cases of lymphedema and 268 cases of hydrocele were reported across 6 counties [1, 7]. By 2015, new confirmed BU cases increased to 105, with 59 cases treated through routine health services [2]. With the global leprosy elimination target of less than 1 case /10,000 people. These findings were reaffirmed in 2016, when the Ministry of Health (MoH) conducted a follow up, concluding endemicity across all 15 counties [2].

Differences in the burden of diseases by county is also evident; for example, in Grand Kru and Grand Gedeh, BU is most frequently reported, whilst onchocerciasis and LF are most reported in Maryland. Conversely, in Margibi and Bomi, hydrocele and yaws are reported most frequently [2].

In recognition of need for effective case management, maintaining the gains of campaigns, and building a system capable of sustaining access to treatment and the achievement of the Sustainable Development Goal of universal health coverage, the Integrated Case Management Program (ICMP) set a goal “to reduce the burden of targeted NTDs to a level that is no longer a public health problem through an integrated control programme, contributing to the socio-economic development of Liberia” [1].

The ICMP fills a gap in community-based NTD prevention, providing linkages for community-based programs lacking household level case management [2]. The programme involves Community Health Volunteer (CHVs) and Community Health Assistants (CHAs), who primarily lead the case searching, referral, diagnosis, and management of the ICMP in the community with SSSDs patients or those displaying symptoms of NTDs [2]. Additionally, the ICMP helps build NTD patients’ capacity to meet their basic needs by removing economic barriers to NTD testing and treatment and improving community awareness of NTDs [2]. Stakeholders across the NTD sector are also advocating for integration due to the opportunities for improving cost-effectiveness [9].

The AIM Initiative is a program of American Leprosy Missions; in 2016, both American Leprosy Missions and Effect: Hope supported the MoH through the NTD department to pilot the ICMP in five counties across Liberia, to gain insights into the program’s effectiveness [2]. The ICMP enables CHAs and CHVs to be directly supervised by the Community Health Services Supervisors (CHSS) [10]. According to the ICMP, CHVs work to identify cases in their neighbourhoods within about five kilometres and report to the health facilities in their reach, through the CHSS. Conversely, CHAs identify cases in the communities further than five kilometres and report to the nearest health facility, also through the CHSS [10]. Both CHVs and CHAs receive training from their supervisors (CHSS) on a wide range of topics relating to NTDs [1, 11]. After a person suspected to have a SSSD reaches the health facility, they are assessed by a health worker who either make a clinical diagnosis or arrange for laboratory testing (typically carried out by the County NTD focal person). Following diagnosis (either clinical diagnosis for leprosy, LF or onchocerciasis, or laboratory confirmed for BU and yaws), the facility health worker initiates treatment with the required medication, under the supervision of the NTD focal person.

### Financing of NTD care

Before 2009, resources and funding sources in Liberia tended to be disease-specific and aligned with each development partners’ workplans and priorities, leading to fragmentation [12]. This system of financing was successfully transitioned to a Health Sector Pool Fund, considered to be more effective [12]. The model was established by the government and its partners in 2008, with support from the Department for International Development, Irish Aid, UNICEF and UNHCR among others [12]. Although it is still largely donor supported (approximately 90% of the health sector resources used in the health sector), the pooled funding model enables the MoH to exercise more oversight of funding prioritization, also enabling coordinated decision making to meet the national health system needs [13]. However, the pooled fund does not include government resources, which typically consists of 11.5% of the national budget across all sectors; with the government contribution going towards personnel administration, accounting for salaries and office space [1, 9, 10]. Government spending on procurement of medicines is minimal, while service funding is mainly provided by donors [13]. There is a reliance on private service provision with 59% of current health expenditure domestic private health expenditure in 2019 [14].

**Fig. 1:**
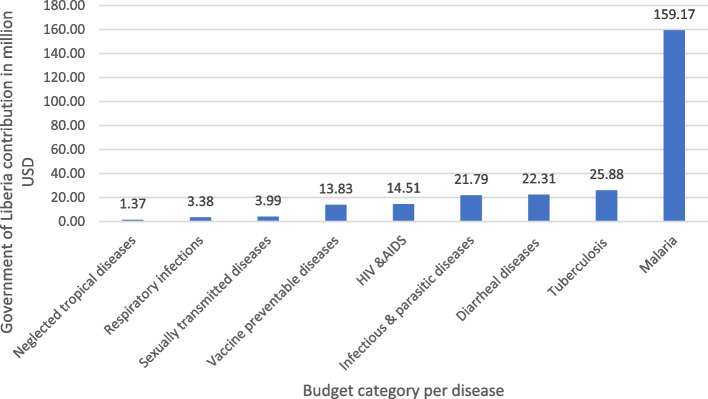
Government of Liberia’s contribution to disease categories for the financial year 2018/2019 (Source: MoH, 2020; MFDP, 2019)

Financing for NTDs is limited in Liberia, being particularly sparse when compared to other national health programmes [1, 9]. As illustrated in Fig. 1 above, the government of Liberia only contributed 1% of the overall expenditure of the NTDs financing in 2018 to 2019, with donor financial and in-kind support being the major contributors towards NTD care [1, 9, 10]. Partners supporting Liberia include American Leprosy Mission and Effect: Hope, involved in Case Management Services; the Liverpool School of Tropical Medicine supporting Preventive Chemotherapy PCT and LF; Sight Savers International who are involved in onchocerciasis; the Global Fund which supports Leprosy drug procurement; and the WHO which supports drug campaigns [1, 9]. Unfortunately, this support is reducing, with funding cuts from UKAID which has implications for the Liberia programme and might undermine progress gained over the years [15].

According to the WHO’s NTD Roadmap for 2030, the goal of an integrated approach is reduced morbidity, disability, and other impacts of skin NTDs [8]. To measure the outcome leading to achieving this goal, the number of countries adopting, localizing, and implementing control of skin NTDs through an integrated approach will serve as the measurement tool [8]. Consequently, the WHO has also called for evidence on approaches to measuring the cost-effectiveness of ICMP [8]. Liberia was the first country globally to introduce an ICMP for the care of people affected by SSSDs. Therefore, Liberia can provide unique insights and learning about this approach, both within Liberia and for other countries also considering this approach.

## Study objectives

This study explores the health financing challenges and opportunities associated with NTD management in Liberia, with a view to strengthen health financing. The study is part of a wider REDRESS research project^1^ investigating how to strengthen integrated case management of NTDs in the country, including a costing of this approach.

## Methods

This section discusses the research methodology. Evidence generated from the literature review process was combined with the primary data from the semi-structured interviews to understand the challenges, barriers, and opportunities for SSSDs financing in Liberia.

### Study design

#### Literature review

A literature search was carried out in March 2020 to identify key texts; Medline and Google Scholar were searched for academic papers, along with websites of relevant organisations, such as CDC.gov for grey literature. The search strategy involved searches on” cost*”,” financ*” and” econom*” in combination with SSSD specific terms:” onchocerciasis”,” buruli ulcer”,” yaws”,” leprosy”, and” hydrocele”. Additionally, we contacted experts in the Liberian MoH and organisations such as COUNTDOWN (is a multidisciplinary research project with disciplines of health economists, lab scientists, parasitologists, and qualitative researchers) to seek further relevant documents. Studies were included if they focused on low-and middle-income (LMIC) contexts, included relevant SSSDs, or NTDs in general, and included information on financing of, or costs associated with, healthcare.

#### Qualitative methods

This study adopted a naturalistic paradigm to give adequate emphasis to the meanings, experiences and views of included participants relating to their experiences with financing and costs experienced relating to providing and seeking care for SSSDs [16] Qualitative research approaches were used because they develop concepts which help to understand social phenomena in natural (rather than experimental) settings, giving due emphasis to the meanings, experiences, and views of all the participants” [17]. This approach was formed of a literature review and primary data collection through semi-structured interviews, which were analysed using the framework approach [14]. The key informant interview (KII) topic guide development was guided by findings from the literature review and previous REDRESS studies [7]. Further participatory approaches were used, with these findings described elsewhere [18].

### Study setting

**Fig. 2:**
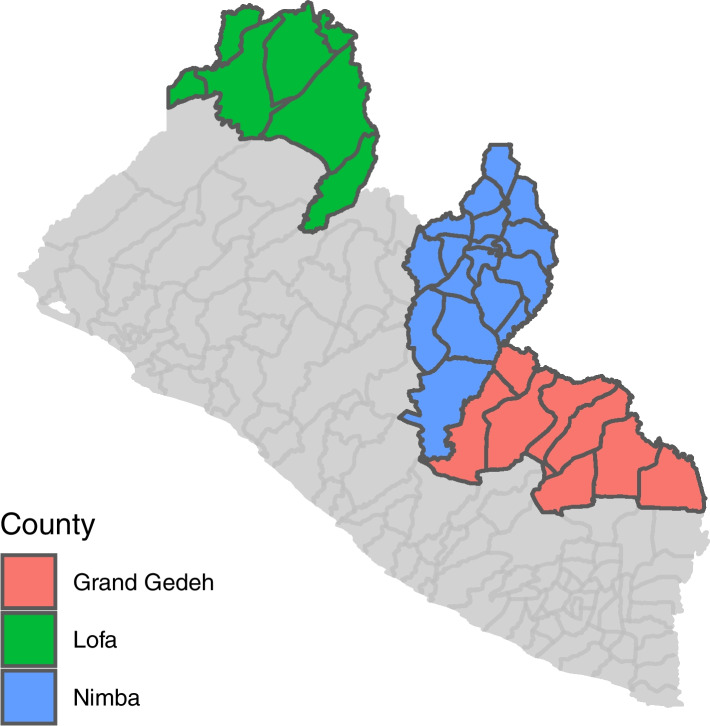
Map of Liberia showing study areas colored in- green, blue and orange

We purposively selected SSSD endemic counties currently implementing the ICMP (Lofa and Nimba), as well as one county providing standard care (Grand Gedeh) as indicated on the map in Fig. 2*.* The two types of counties were chosen in order to allow comparison of health financing between those implementing and not implementing the ICMP.

### Study sample and sampling method

There were 102 participants, of which 86 were drawn from the county, district, health facility and community levels and 16 were drawn from the national level. Participants were purposively selected based on their specialized roles and experience in working with NTD patients across different levels in the study area. All participants were asked financing questions as part of a multidisciplinary research. See Table 1 for a summary of participant characteristics.

**Table 1:** Participant characteristics

Participant category	County level participants		National level participants
	Implementing ICMP (Lofa and Nimba counties combined)	Not implementing ICMP (Grand Gedeh County)	
County Health team members (e.g. pharmacists, clinical directors, etc.)	12	12	0
County Referral Hospital Level	5	5	0
District/Health Facility Level	19	18	0
Patients, caregivers, Traditional headers and Community leaders	9	8	0
National policymakers (e.g. Directors, Chief Pharmacist) etc.	0	0	16

### Data collection

We recruited and trained four local data collectors in the Lofa and Grand Gedeh counties, who worked with four research fellows to collect data at county,^2^ district,^3^ health facility and community levels. Data collectors were trained for 2 days inclusive of 1 day for piloting the KII topic guide with the research fellows. Meetings were also held with authorities of county and district health stakeholders, where we provided information on the study objectives and activities, prior to deployment of data collectors from October to December 2020. Our data collection team interviewed members of the County Health Team (CHT), District Health Team (DHT), Hospital and Health facility management system, and community leaders from Grand Gedeh, Nimba, and Lofa counties. To support trustworthiness during data collection, we conducted a daily debriefing meeting with the research fellows and the data collectors, to discuss daily field activities and mitigate potential challenges encountered.

After the county level data collection process, we commenced the national level data collection with the four research fellows serving as data collectors from January to March 2021. The participants for the national level data collection included directors, administrators, supervisors, and pharmacists, who were purposively selected based on their experience providing guidance and leadership in development of programs, standards and operating procedures for both national and county levels operations. The topic guide covered the larger research body of REDRESS, including clinical epidemiology and laboratory systems strengthening and human resources for health management and patient centered approach, as well as questions regarding health financing for SSSDs.

### Data analysis

Verbatim manual transcription was conducted by trained translators form University of Liberia - Pacific Institute for Research & Evaluation (UL-PIRE) immediately following interviews, with randomly selected transcripts checked for quality assurance purposes. An anonymous identifier code was developed and used during data collection and transcription to identify participants’ categories and roles, whilst maintaining confidentiality. Next, we conducted analysis on NVivo 12 using the thematic framework approach [19]. The team of researchers from UK and Liberia inductively developed coding frameworks and themes categorizing participants responses from the transcripts. We further developed nodes, daughters’ nodes and created chart summaries. The charts and summaries were used to produce summaries and special quotes that were furthered developed into narratives representing themes.

### Ethical considerations

The study received ethical approvals from both the Liverpool School of Tropical Medicine Research Ethic Committee in the United Kingdom (ethic # 20-040) and UL-PIRE’s Institutional Review Board in Liberia (ethic # 20-07-221) in March and April 2020, respectively.

### Study limitations

The main limitation for this study is the lack of more detailed data on systemic and households’ costs due to the COVID-19 pandemic, which are set to be addressed in the next phase of the project, for which this paper is preparatory. As part of a larger body of research, the efforts planned for data collection was for both local and international peer researchers to go into the field. However, some limitations brought by the COVID-19 Pandemic and associated travel restrictions hindered our international researcher’s participation into field data collection.

## Results

The results have been reported using themes which are structured into five main sections, with section one presenting evidence from the literature highlighting the research gaps which this paper addresses and the remaining sections exploring findings from the KIIs. Section two explores the views of participants on financial decision making processes at the national and county levels in Liberia. While section three explores the roles within financial decision making at different levels. Section four presents challenges with financial processes and planning at each level, whilst section four discusses out-of-pocket (OOP) costs. Section six details opportunities and recommendations for NTDs financing in Liberia and finally, section seven colligates findings from the evidence summary of the literature in LMICs and Liberia specifically.

### Literature review: evidence summary from LMICs and Liberia

#### Economic evaluations and modelling of SSSDs interventions

The predominant source of evidence from economic evaluations comes from two systematic reviews, which cover onchocerciasis and lymphoedema interventions [20, 21]. One Individual study was also identified for yaws and hydrocele interventions, although no economic evaluations were found for the latter [22]. No studies reported on BU interventions and also, there were no economic evaluations for integrated case management.

The current literature lacks evidence on SSSDs costing, partially since the term SSSDs is relatively novel, although the evidence for MDA is also scarce [23]. Even when SSSDs are grouped with NTDs, there remains a gap in costing of services of the various pathways for seeking care, such as the direct and indirect costs associated with care seeking, feeding for both patient and caretaker, transportation, in-patients incur costs on toiletries and food items (daily food rations may not be enough for patient and family) [24]. Patients also underwrite costs of medications when there are stockouts and those admitted to in-patient care lose out on potential earnings, with the length of stay ranging from weeks, typically in LF patients, to years in individuals with leprosy [20, 24]. Even after being discharged, patients underwrite the costs associated with traveling back home, highlighting a cost implication which has not been costed in Liberia.

To the best of our knowledge, there have been no comprehensive costing studies conducted for SSSDs or NTDs in Liberia except for one study done by Popovic, et al. (2017) that reviewed Marginal Budgeting for Bottleneck (MBB), Core+ and the One Health tool as costing tools that have been used in Liberia for core services, such as basic health packages [25].

Across other LMIC settings there was only four papers of economic evaluations on SSSDs, and of these, only one adopted the patient perspective [24]. Also, whilst these studies present evidence of available Economic Evaluations for lymphoedema and onchocerciasis, there is a lack of evidence regarding yaws, hydrocele and BU, as illustrated in table three [20–22, 26].

Crucially, we found no studies which investigated how the integration of SSSD and other NTD programmes affect programmes’ costs and cost-effectiveness. A paucity of information on productivity losses experienced by informal caregivers.

#### Key findings for Liberia

In Liberia, a full package of costing for the health system inclusive of costing of NTDs or specific skin diseases, has not been conducted [2]. To the best of our knowledge, there is no literature on financing or costing of NTDs or SSSDs, and information on costing projects in Liberia is scarce. According to the literature, there is currently no costing being conducted for SSSDs in Liberia up to the time of writing this evidence summary. However, it is unclear if the lack of literature is due to a lack of costing being conducted in Liberia, or rather a lack of reporting [7].

Costing tools previously piloted in Liberia include CORE Plus by MSH in 2009; the WHO’s MBB tool for health and nutrition interventions of the Millennium Development Goals [25]. Also piloted, were USAID’s One Health tool used for HIV & AIDS interventions and their database developed for costing services to support policy and decision making at the MoH [25]. Among these piloted tools, the MoH has selected the USAID supported database as the accepted tool for costing services in Liberia. The main reported drawback of the tool is that it is not web based [7]. However, for uniformity purposes, the MoH has recommended the use of this database costing tool for all partners supporting the Ministry through implementing costing services in Liberia [7].

### Qualitative findings

#### Financial decision making processes

Many participants highlighted that the Ministry of Finance and Development Planning produce the projection for line Ministries and Agencies and bring it to the national legislature for approval. The approval budget is then presided over by the Ministry of Finance and Development Planning and the MoH like other ministries are required to make requests based on priority activities and the availability of funds.

#### National level

Six national managers stated that financial decision making usually occurs in sector and strategic coordination meetings, among others where local county and district health authorities are not represented. However, other managers highlighted that the financial decisions are made internally by departments, before going to the general coordination meeting.

*“OK, government’s own competing priorities exist, so, therefore, department directors usually call meeting for us to internally agreed on some decision before going to general coordination meeting” National level key informants, 029, Monrovia*

#### County level

Respondents from all levels said that financial decisions at the county and district level are limited in many ways for the general health system. For example, in procurement of goods for the health system at county level, there is a benchmark of not exceedingly more than US$10,000.00 per quarter when procuring goods for all programs, inclusive of the NTDs program. Seven participants explained that financial decisions are made through the different programs supervisors who usually participate in fiscal planning and forecasting meeting on a yearly basis at the county level. However, whist these forecasts are sent to the national level, they are not binding, and may not be used by the national office. Rather, other program financing instructions are sent from the national level to the county with an approved budget and direction for the usage of the approved budget. Four county level participants expressed that the county authorities have no power to alter the financial decision made at the national level, even when it does not align with priorities. A similar situation was described in both ICMP and non-ICMP counties.

*“Our plan is sent to national level. If it is sent, national too and her partners consolidate all those plans. For NTD for example, all those partners that are supporting NTD activities will say I can support this one. National level will plan and communicate their plan. And it comes with budget line which at county level you cannot divert so easily. So, if you will divert it, it must be communicated. So national level too will send a budget line and you go straight by the implementation of what the budget lines states. … .” County level key informant 012, Grand Gedeh County*

“*Financial decisions are made through the management of the Central office. Before finances are provided usually the County makes their request to national. So, the request national will look at it and either leaves it like that or adjust based on the availability of resources. And when finances come, they come with template on how it should be utilized. So, the leadership along with the NTDs team or surveillance team as well as others make decisions based on the guide that is provided for implementation by national. So, this is how decisions are made*” *County level key informants, 018, Lofa County*

Overall, financial decentralization for NTDs is not yet achieved, but for other budgets such as Malaria control program, Community Health programs, Health Promotion, Non-Communicable Diseases and the Tuberculosis (TB) control program decentralisation has been achieved. Our findings suggest that financing (including information) for NTDs remains centralized and largely donor driven.

### District and health facility levels

Almost all the Officers in Charge and District Health Officers interviewed confirmed a lack of power to decide or participate in financing discussions and decision making at the district and facility level. They explained that they only receive supplies and materials upon request.

*“At this level, we receive only supplies of materials do not cash or making financing decision. When our materials are finished, we can write the county and the county write national or the county supply us what we want when they have the money to buy them” District level key informant 021, Grand Gedeh County*

In summary, national level actors are charged with an authoritative fiscal planning, while county level actors inform fiscal planning by providing suggestions to national level for inclusion where applicable. Conversely, district and facility level decision making is minimal.

#### Financial decision making

This section explores the different roles within financial decision making, in terms of key actors involved, funding sources and donor contributions.

#### Who is involved?

Participants from all levels agreed that financial decisions for general health programme are being made through planning with partners and the MoH, with NTD financing decisions made through the NTDs ICMP. However, most of the respondents noted that financial decisions are highly directed by program and donor who provide the funding for the implementation of the program.

*“Thank you very much. So, the roles of most of our partners were incredibly positive, they served primarily as funding partners and facilitators. They helped to provide pool of information resources that helped to inform our plan but they did not direct what the plan could be made of, they did not direct what were the priorities; but rather, they provided that support to the ministry of health while the ministry of health and Liberians led the development of their own plan, making decision priorities through sector meetings and strategic coordination meeting … …” National level key informants 008, Monrovia*

Other managers from the national level stated that financial decisions require the approval of the MoH and signed memorandum of understanding between the MoH and partners, such as ACTs (formerly MAP) who serves as an independent financial body to manage and report on partners resources on behalf of the MoH.

*“Alright so like the financing of drugs, the procurement of drugs, and medical supplies for NTDs interventions, the approval has always been the ministry of health even though the case management comes from and manage by ACTs formally MAP, but whatever request, budgets, memorandum of understanding can be signed between the ministry of health and the partners and then of course the third party ACTs, so whatever implementation that’s supposed to be done here that request is being approved by the office of the chief medical officer and then before it is being implemented, be it request of procurement of drugs and medical supplies” National level key informant, 007, Monrovia*

#### Sources of funding

As previously observed, funding for NTD services within Liberia is highly donor dependent in both case management and non-case management counties. However, there were more gaps described in non-case management counties, for example Grand Gedeh compared to Lofa.

According to almost all national level participants, the government’s funds and contributions to the health sector been scarce, except for malaria programmes where they provide approximately 60% of the cost to run malaria programs and purchase drugs. More than five participants asserted that for other diseases including NTDs, the government contribution goes towards personnel and office costs, contributing only 1% of the overall program cost for NTDs services nationally. The cross-reference Table 2 above shows the 2018/2019 Fiscal year budget and expenditure in percentage point of government contributions to health system strengthen in Liberia.

**Table 2:** Summary of the economic evaluations in LMICs reported in the literature review

Condition	Intervention(s)	Studies Reporting	Best Cost-effectiveness	Best Cost-Benefit
Hydrocele	Surgical interventions	Yellu (2010)	NA	NA
Yaws	Sequential testing: treponemal RDT before a trep/non-trep RDT	Fitzpatrick 2017	ICER is US$ 58 (42–103) per correct diagnosis gained	NA
Onchocerciasis	Ivermectin MDA (OCP, APOC, or Annual MDA)	Turner 2019 (plus 8 CBA and 7 CEAs included in this); Kim 2015 (non-E.E.s reporting economic info on interventions: Boussineq 2018; Turner 2013; Verver 2018 – see text below)	See Table 2 of Turner 2019 e.g. $13.4 per healthy life-year added (Benton 1998, APOC, cost horizon 1996-2017), $7 per DALY averted (Remme et al. 2006; APOC; cost horizon 25 years)	See Table 3 of Turner 2019
Lymphoedema	MDA (drug combinations unclear) (e.g. GPELF)	Gedge 2018 (and 12 E. Es included in this)	See Table 1 of Gedge 2018 e.g. $5.90 per DALY averted (Ottesen 2006, Annual MDA, 30 year time horizon).	See Table 2 of Gedge et al. 2018

Ten national level participants asserted that government funding contributes towards human resources and salary payments through the government’s Civil Servant payroll system, with the offices and government buildings being used for health services.

*“Well, as I told you, with Human Resource, the salaries are paid the government, the office space and building but all other expenditure, 100% depend on partners whether preventive chemotherapy, whether case management, everything depends on partners” National level key informant, 003, Monrovia*

#### Donor contributions

In this section, our findings represent views about donor funds and in-kind support (not Performance Based Financing). Some of the National level managers expressed that the donor funding or in-kind support consisted of support to standalone programs like HIV/AIDs or TB/leprosy, among other programs.

**“***Umm, first of all, you know the ministry activity is not very programmatic if it comes to the budgeting aspect, so you will not find most of these things being flag out as a standalone activity, except for those that have been donor focused like the HIV and AIDs, like TB and leprosy, like TB and malaria those are things that stand alone because they have particular commitment and agreement with the ministry, but other than those you have all other services being done generally from the government perspective, except for donor in kind support or donor commitment to different focused programs that are donor specific in kind support or direct funding” National level key informants, 030, Monrovia*

Other national managers also highlighted that the donor funding or in-kind support consisted of medicines and medical products (Preventive chemotherapy drugs) donated by pharmaceutical companies, with distribution funded through programs like that of LSTM, SCI and Sightsavers.

Leprosy Multidrug Therapy drugs are donated by Novartis, while American Leprosy Mission has funded laboratory reagents and deploys Gifts-In-Kind shipments, including other medicines and products. This illustrates that donor financing and in-kind contributions account for most medicines and consumables for Liberia’s NTD program.

*“Yes, sure but we do provide services and bulk of those in-kind support and funding come from partners and you know, partners donate them us, included are those medical, medicines and medical products … …” National level key informants, 001, Monrovia*

Other national managers stated that the donor funds or in-kind support finances the $5 package support^4^ to CHAs and CHVs who identify potential cases, with remaining funding going towards personnel salaries.

### Challenges with financial processes and planning

Participants highlighted quantification and planning, inadequate government involvement and donor involvement as key challenges.

#### Quantification and planning

Quantification of goods and budget estimating were specific challenges described, given the high donor involvement and limited government role currently. National Managers expressed that quantification was done with partners in quantification meetings with minimum government participation.

*“We usually do quantification in our quantification meeting with all the partners in attendance. Although, the government has limited role and county pharmacists from the 15 counties cannot be in all the meeting but we something look at their report to know the previous consumption level” National level key informants, 008, Monrovia*

Our findings demonstrate that decision makers for NTDs are not being represented during quantification, with attendees unaware of the actual supply needs for the NTDs program. National level participants stated that there are no NTD representatives in quantification meetings, with projections for NTD drug needs based on assumptions of attendees. Attendees usually includes members of the supply chain, health promotion and pharmacy departments. This was echoed by managers at the district and facility levels, who highlighted financing discussions and budgets as challenges, owed to their limited or non-existent decision-making power.

To further complicate the quantification decision making process, participants highlighted that partners involved in the normal protocol for drug quantification are not the same as the NTD partners, leading to frequent stockouts due to inaccurate estimates.

*“I have not seen NTDs representative from the department during quantification meeting like Pharmacist or so. We have been looking at the previous supply records and assumptions. I am sure they will be represented in future quantification. I think it is a good idea to have someone representing them like pharmacist or so” National level key informants, 010, Monrovia*

#### Limited government involvement in NTD financing

Participants across all levels agreed that there is a lack of budgetary allocation for SSSDs financing at the national and county level, with support limited to MDA and no government allocation towards case management of people affected [1, 20]. Most district level participants also noted inadequate funding at the district and facility level,

*“With regards to SSSD, there is no budget allocated at national and the county levels. Yeah, but usually what happen, we only receive budget when it comes to Mass Drug Administration (MDA), to distribute drugs throughout the entire country for everyone especially ages from five to fourteen” county level key informant, 005, Lofa County*

While five other County level managers stated that the salary payments by the government are marked by huge discrepancies in salaries among clinicians which is yet to be addressed by the Civil Service Agency and the Ministry of Finance. Moreover, participants across all levels agreed that stockouts of medication and supplies at facilities, combined with delayed salary payments are driving low staff attendance and retention. District level participants suggested activities for addressing these challenges, including focusing on improving personnel retention and management.

*“Yes, the government paid salaries which have had discrepancies with payments among clinicians which is beyond our control; from civil service and the Ministry of finance, we have been talking about it and engaging them but no result. I am a nurse, and you are nurse maybe I make US$140.00, and you make US$250.00 and the both of us are nurse” County level key informant 022, Grand Gedeh County*

County level staff expressed different views; for example, one participant identified that government funds consisted of fuelling the ambulances, while another stated that funds are directed at the county level, with special instructions on its implementation, such as for fuelling the generators, or gasoline for motorbikes.

#### Donor support and involvement for NTDs

Donors coordinate around the integrated case management and supply different goods and support other parts of the process as part of that in the ICMP counties with noticing of once sever frequency of drug stockouts per month as compared to non- ICMP where supply also depends on NGOs and other donors with limited government support but with big gaps and more than three times sever frequency of monthly drug stockouts.

### Out-of-pocket costs

Participants across all levels agreed that limited funding has implications for the quality of services, one of which is that patients are forced to pay OOP costs for care e.g., blades for diagnosis, prescriptions if stock outs occurs and transport costs.

“*When the patient is discharged, remember you took them to the hospital through the ambulance and the patients themselves have to take care of the issue of food, toiletries, and other things such as accommodation for caregivers. When the patient is discharged, the patient supposed to come home, who takes care of that transportation cost to come home? Is the patient” Health facility level key informant 019, Grand Gedeh Count*

The greatest challenge highlighted by patients and community level participants were out-patient expenditures on transportation (with patients often refused motorbikes or overcharged, due to fear and stigma), medication and food/toiletries if in-patients.

Conversely, all county level managers stated that OOP costs usually consist of purchasing antibiotics, wound dressing materials and gauze during stockouts at the county and facility level. This was the most expressed view.

*“What is done for patient, we just do our ordinary antibiotics that may be available. But sometimes we tell patient to buy these things. Most especially when the dressing materials from the county level are not available. We tell patient please get your gauze despite the needed gauze from the NTD belt is not there so that we can use the initial dressing till the NTD gauze can come or until they call their county NTD focal person can come” County level key informants 014, Grand Gedeh County*

#### Who is responsible for paying out-of- pocket costs?

All patients, household heads and community leaders agreed that communities share costs for transportation/support for food. This was the most expressed view.

*“Mainly it is the patient that bears the cost during stockout of medications at the facility and then the patient came to seek care, you will find out that the patient will be given prescription to go and purchase their medication. And whenever patient do not have money, it become serious problem for the patient” Health facility level key informant 019, Grand Gedeh County*

District and facility level managers stated that family members or caretakers bear the cost for OOPs, with others reporting that costs are sometimes covered by advocates, through health worker appeals.

“*It is the family that bear the cost. Like I said, it is family because if the family does not have money, then the advocates in the community, because if somebody come with NTDs condition and they are treated and there is no funding for them to go back home they cannot stay in the hospital. You go and appeal and advocate for them and say, oh, we got client and I am finished with their treatment, so we want them to go back home. Sometimes and also, we negotiation alternatives like if any car is going in the same direction, we can talk to them to help the person by giving them lift in their car … …” Community level key informant 020, Lofa County*

Six community level participants expressed different views from other community leaders and household heads. For example, four participants stated that OOP costs often fall on the shoulders of patient’s relatives and family members. While two participants stated that the burden is usually directed to the clinicians providing the services, to the extent that sometimes they are forced to use petty cash authorized by the CHO (County Health Officer) to transport patients home.

*“The costs are sometime shouldered by the patients’ relatives or the clinician providing the service through CHO bears the cost, if petty cash is available for help” Community level key informant 021, Lofa County*

#### Financial implications of out-of-pocket payments for people affected and their families

Three community level respondents highlighted how OOP expenses force individuals to weigh up the cost of seeking care and loss of earnings with the benefit of receiving care.

“*I have to encourage my uncle and took him to the hospital. he did not want to go to the hospital because the hospital is far from our town, and he thinks that if he goes there, he will miss on the opportunity to get our daily meal through fishing and farming and doing daily work for other to earn food money …” Community level key informant 022, Grand Gedeh County*

### Opportunities and recommendations for NTD financing

Our results demonstrate that whilst NTD services are provided in the ICMP counties, the quality of these services is undermined due to inadequate funding, leading to stockouts, etc. Respondents suggested different avenues for generating additional funding to strengthen the quality of care, including budgeting at county level, public private partnership, county social development funds and reintroduction of user fees.

At present, NTD care services include free management services for NTDs, such as screening, lab testing/specimen collection and diagnosis, medication, and complication management. These services are usually free in the five piloted ICMP counties, compared with non-ICMP counties where only annual MDA and standard care are provided free of charge. Moreover, complicated cases from ICMP or Non-ICMP counties are often referred to the referral hospital or to Ganta Rehab (Leprosy rehabilitation centre), with patients and care takers from ICMP counties provided with ambulance transportation, medication, and treatment free of charge.

Eleven key informants highlighted opportunities for NTDs and general health system financing in Liberia. One participant suggested that the reintroduction of fees for service or cost sharing could help solve the stockouts observed over the years.

“*The reintroduction of payment system as the fee for service or cost sharing will help solve the stockouts problems, where minimum fee is charge for the service” County level key informant 014, Grand Gedeh County*

While several participants viewed private sector partnership as an opportunity for NTD financing in Liberia, others emphasized that increasing awareness of NTDs/SSSDs, perhaps via radio, will call the attention of private investments in financing these diseases.

Others emphasised opportunities for NTD Financing through the County Health Board, which is chaired by the political leadership of the county (Superintendent), someone perceived as having the political power to influence resource allocations for development financing.

Two county level managers stated that an additional opportunity for NTDs/SSSDs financing might be through the county social development funds^5^ and individual citizen donations.

*“As an innovation, let start thinking about using the county social development funds and also individual contribution to the financing of NTDs/SSSDs in our country since it is affecting our people” County level key informant 028, Nimba County*

## Discussion

Our study provides insight into the current global and national gaps in evidence for costing of NTD and SSSD services, with key findings from primary data highlighting key challenges and opportunities for financing for NTDs in Liberia. Challenges include the minimum role played by the government in NTD Financing, decentralized financing and decision making, the causes of NTDs medication stockout due to the exclusion of the NTDs program from the general quantification of the health system. Opportunities include mobilising part of the social development fund to finance the health system and cost sharing with the government.

### Decision making and budgeting

Our findings provide evidence of a top-down budgeting approach, although not only specific to NTDs. Financing and the health financing model shows limited government role in quantification and a lack of financing information for the district and health facility levels. The government of Liberia, through the Ministry of Finance and Development Planning, the National Legislature and the MoH, are responsible for allocations, approvals and providing instructions for budgetary countywide implementation and the use of resources. Low-priority areas, with financing failing to reflect the financing needs of the district or community. This type of financing does not align with lower-level funding needs, rather what is generally perceived as the health system’s needs [22] and shows divergence from what is reported in the literature reviewed.

Our findings also shows that partners’ funding decisions were made in partnership meetings, committee meetings and based on the MoH policies and financing needs. For example, the NTD program’s supporting partners depend on the ICMP in deciding what component of the program they will direct their support consistent with the finding of Kollie, K, Siakeh, A. et al., 2021 [27]. This finding is also in line with the WHO’s NTD roadmap, pillars on “Change operating models and culture to facilitate country ownership” [28]. Decisions are further communicated with the Ministry through partnership meetings, specialized meeting or specially arranged meetings for endorsement, acceptance, or approval by the ministry through the department compared to non-ICMP counties, where these opportunities do not exist. The implications of this strategy for county and district level implementers are that local priorities are not influencing the budget during the implementation at the county and district levels.

### Type of funding sources

Our findings shows that there are limited funding sources for the financing of the NTDs program as compared to other similar programs. The literature has mixed reactions on funding sources but mostly limited sources as well [23, 27]. This is particularly true in cases where there is absolutely no financial contribution from the government to the NTDs program. Consequently, NTDs program continuation is highly dependent on partners’ ability to obtain other donors’ support. With the changing funding environment, such as the reduction in UKAID, this creates uncertainty surrounding the sustainability of NTD service provision and threatens the progress already made [15]. The likelihood that the program could close when this type of funding sources is not improved is considerable if all the partners draw down in future. Additionally, the government’s financial leadership and ownership of the program expressed through a minimum financial contribution will motivate partners and assure them of the program sustainability. It will also improve the sustainability of the program financing and may open a door for more new sources of funding.

### Political priority for NTDs

Our qualitative findings demonstrate that NTD programs are not a financial priority of the government, with the ministry funds not reflecting or considering NTDs. This is supported by the literature from other LMICs [20, 21, 23, 27]. However, one could argue that the government has shown some prioritization of the NTDs program through policy with the development and approval of the piloted ICMP. Although the case management program was developed through the financial sponsorship of other partners (AIM Initiative), with the MoH playing a leadership role. Consequently, there is no strong political will for NTDs financing by the MoH, as compared to financing for other diseases. Political priority for NTDs would require budgetary prioritization and the elevation of NTDs in political and national speeches as with HIV and AIDS, Malaria, Ebola and COVID-19 which have all been publicly discussed among politicians and policymakers of the country. Knowing that currently there exist huge prevalence rate for example BU prevalence rate according to WHO,2015 survey across all 15 counties of Liberia shows a national prevalence estimated at 0.82/10,000 people [2].

### Patient pathway barriers: financial

Our findings show that SSSD patients are faced with both financial and non- financial barriers, such as transportation due to the distance from the health facility, feeding for patients and caregivers, accommodation for caregivers and non-financial barriers such as stigma, rejection from family members and loss of relationships. Therefore, if we are to successfully care for people affected by SSSDs, policy must incorporate patient perspectives; this could help to address patient’s pathway barriers, aligning with the findings of Sunyoto et al’s 2019 systematic review, which emphasises the need for patient non-medical needs such as food, transportation, accommodation, etc. [29].

According to WHO’s 2017 global statistical, 40% of NTD affected people are found in Africa, equating to approximately 600 million people affected [28]. They stipulate that eliminating the disease will require implementing simple and affordable to save the lives of millions of people [28]. In Liberia, the ICMP has successfully rolled out MDA to 2.5 million people with about 460,000 people in need of treatment [2, 23, 30, 31].

Delivering treatment to this group will require not only a supply side cost-effective integrated plan, but incorporation of a demand side approach through addressing financial and non-financial barriers associated with the involvement of patients’ family and relatives who provide care, through actively engaging them in the design and implementation of policies that aim to address their needs. For example, since SSSDs are generally found in poor communities, if affected people were the only bread winners for their families, they would be more concerned about their family food needs and survival than seeking medical care at health facilities consistent with existing studies [24, 29, 32, 33]. Our results also revealed that patients delay care seeking to obtain food and other necessities for their family, which is in keeping with findings from other studies which highlighted the need to consider loss of wages prior to seeking care for SSSDs.

### Recommendations for further work

Building on the literature review findings, the authors recommend the following areas be addressed in future research. Firstly, the knowledge gap in the costing of SSSDs care in Liberia from societal and patients’ perspective must be addressed. Cost-effectiveness analyses of SSSD interventions in Liberia should also be conducted to identify cost drivers and incremental cost differences, to help guide SSSD policy and programs. This preparatory study for the wider REDRESS research project will contribute towards filling some of these critical evidence gaps.

## Conclusion

Our findings demonstrate that there is very limited evidence on health financing of SSSDs in Liberia and more widely in the region. NTDs programmes face low prioritisation by the MoH and remain heavily donor dependent. The study also shows that households face large costs in accessing care, which are exacerbated by stigma. Therefore, it is, important to conduct additional economic evaluations to support more effective care packages, which also shift the costs and pool risks more effectively for affected populations, which are often amongst the most disadvantaged populations, living in rural areas of high poverty.

## Data Availability

The dataset used for this study are available based on request in line with the UL-PIRE and LSTM protocols.

## Abbreviations

AIM: American Leprosy Mission
BU: Buruli ulcer
CHA: Community Health Assistant
CHSS: Community Health Service Supervisors
CHV: Community Health Volunteer
ICMP: Integrated Case Management Program
KII: Key informant interview
LF: Lymphatic filariasis
LMIC: Low and middle income country
MBB: Marginal Building for Bottleneck
MDA: Mass Drug Administration
MoH: Ministry of Health
NGO: Non-governmental organisation
NTDs: Neglected Tropical Diseases
OOP: Out-of-pocket
SSSDs: Severe Stigmatizing Skin Diseases
TB: Tuberculosis
UL-PIRE: University of Liberia – Pacific Institute for Research & Evaluation
WHO: World Health Organisation

## Footnotes

1 REDRESS Project website link https://www.lstmed.ac.uk/projects/redress-reducing-the-burden-on-severe-stigmatising-skin-diseases

2 Average population for county is 160,000 people

3 Average population for districts is 32,000 people

4 CHVs are provided with $5 incentive for each confirmed NTD case they identify.

5 Social Development Funds – are the resources paid by concessionaries companies to the county in the form of social corporate responsibilities. These monies are pay annually on a regular basis.
